# Tumor-derived cell-free DNA detected in cerebrospinal fluid enables minimally invasive profiling of pediatric brain tumors

**DOI:** 10.1172/JCI197391

**Published:** 2026-06-15

**Authors:** Liana Nobre, Yoshiko Nakano, Ian Burns, Robert Siddaway, Michal Zápotocky, Monique Johnson, Mansuba Rana, Cyril Li, Rodney K. Lyn, Richard Yuditskiy, Michelle Ku, Javal Sheth, Adrian B. Levine, Cody L. Nesvick, Anirban Das, Chantel Cacciotti, Shayna Zelcer, Seth A. Climans, Maria MacDonald, Logine Negm, Jiil Chung, Julie Bennett, Andrew Bondoc, Jim Loukides, Lucie Stengs, Melissa Edwards, Eric Bouffet, Vijay Ramaswamy, Anthony P.Y. Liu, Annie Huang, Ute Bartels, Peter B. Dirks, Uri Tabori, Cynthia Hawkins

**Affiliations:** 1 Division of Hematology/Oncology (iHOPE), Stollery Children’s Hospital, Department of Pediatrics, University of Alberta, Edmonton, Alberta, Canada.; 2 Arthur and Sonia Labatt Brain Tumour Research Centre, The Hospital for Sick Children, Toronto, Ontario, Canada.; 3 Institute of Medical Science, University of Toronto, Toronto, Ontario, Canada.; 4 Division of Hematology/Oncology, The Hospital for Sick Children, Toronto, Ontario, Canada.; 5 Department of Paediatrics, The Hospital for Sick Children, Toronto, Ontario, Canada.; 6 Department of Pediatric Laboratory Medicine, The Hospital for Sick Children, Toronto, Ontario, Canada.; 7 Department of Paediatric Haematology and Oncology, Charles University, Second Faculty of Medicine and University Hospital Motol, Prague, Czech Republic.; 8 Department of Laboratory Medicine and Pathobiology, University of Toronto, Toronto, Ontario, Canada.; 9 Division of Neurosurgery, The Hospital for Sick Children, Toronto, Ontario, Canada.; 10 Children’s Hospital London Health Sciences/Western University, London, Ontario, Canada.; 11 Department of Clinical Neurological Sciences, Schulich School of Medicine and Dentistry, Western University, London, Ontario, Canada.; 12 Department of Oncology, London Health Sciences Centre, Schulich School of Medicine and Dentistry, University of Western Ontario, London, Ontario, Canada.; 13 Division of Medical Oncology and Hematology, Princess Margaret Cancer Centre, Toronto, Ontario, Canada.

**Keywords:** Genetics, Neuroscience, Oncology, Brain cancer, Drug therapy, Molecular diagnosis

## Abstract

**BACKGROUND:**

Liquid biopsy has emerged as a minimally invasive method for tumor diagnosis, monitoring, and therapeutic guidance. For CNS tumors, cerebrospinal fluid (CSF) provides a reliable and accessible source of tumor-derived cell-free DNA (ctDNA).

**METHODS:**

This study evaluates the clinical utility of CSF liquid biopsy in a real-world prospective setting. A total of 148 CSF samples from 120 patients underwent molecular analysis using droplet digital PCR (ddPCR) and/or next-generation sequencing to detect mutations, fusions, copy number alterations, and mismatch-repair deficient signatures (MMRDness). Samples were collected via lumbar puncture (*n* = 82; 45% ctDNA positive) or from ventricle sources at the time of surgery or through shunts (*n* = 66; 65% ct DNA positive).

**RESULTS:**

Overall, ctDNA was detected in 54% of samples with higher detection in high-grade gliomas at diagnosis (100%, 1 sample equivocal) compared with low-grade gliomas (50%). Among low-grade gliomas, ctDNA detection was higher in disseminated cases (80% versus 43%) and from ventricular versus lumbar samples (56% versus 38%).

**CONCLUSION:**

Liquid biopsy distinguished relapse from second malignancy and serial sampling demonstrated the potential for ctDNA levels to track treatment response and disease progression. In patients with MMRD tumors, high MMRDness score from ctDNA supported active disease. These findings demonstrate that combined liquid biopsy assays facilitate diagnosis, monitoring, and personalized treatment decisions, offering a viable alternative to invasive surgical biopsies in pediatric CNS tumors.

**TRIAL REGISTRATION:**

None.

**FUNDING:**

Proof of Principle Grant from The Hospital for Sick Children; The Canadian Institutes of Health Research; The Canadian Cancer Society; The We Love You Connie Foundation; Garron Family Cancer Center at SickKids; SickKids Clinician Training Program; Ben Stelter Foundation through the Women and Children’s Health Research Institute; Jeffrey Brock Cancer Genetics Research Fellowship; Garron Family Cancer Center Research Fellowship/Scotiabank Clinician Scientist Fellowship; Atrium/CMCC and Hold’em for Life Oncology Fellowship; Tokyo Children’s Cancer Study Group Scholarship of the Gold Ribbons Network.

## Abstract 2

<a></a>This study highlights the clinical utility of liquid biopsy in a large cohort of pediatric patients with CNS tumors.

## Introduction

Central nervous system (CNS) tumors are the most common cause of cancer-related death in the pediatric population (1). Recent substantial progress in the molecular characterization of these tumors has enabled more accurate diagnosis and prognostication, in addition to ushering in a new era of targeted therapies. These advances have led to improved outcomes and have reduced the risk of severe adverse events in patients with CNS tumors (2–6). However, obtaining tumor tissue for analysis is not always possible and is accompanied by inherent risks that can lead to significant morbidity. Therefore, in clinical settings, therapy is sometimes initiated based solely on clinical and radiological features, or watchful waiting is favored. Current imaging methods for assessing treatment response and disease surveillance in CNS tumors often lack the sensitivity to detect minimal residual disease or early recurrence. Additionally, repeat biopsies pose major challenges due to their invasive nature and associated risks, limiting the ability to obtain detailed information on tumor evolution, development of therapy resistance, and identification of new therapeutic targets. These limitations underscore the need for alternative approaches, such as liquid biopsies, which offer minimally invasive and dynamic molecular profiling.

Liquid biopsy, through the analysis of tumor material from body fluids such as blood or cerebrospinal fluid (CSF), can be used for tumor identification and diagnosis. It also has the potential to provide greater sensitivity for monitoring treatment response and detecting minimal residual disease when compared with conventional imaging and biomarker analyses (7, 8). Currently, several liquid biopsy tests targeting circulating tumor DNA (ctDNA) are clinically approved. However, the majority were developed for the detection of therapeutic targets in adult solid tumors using blood samples (9). For CNS tumors, it has been shown that CSF is a superior source for liquid biopsy when compared with blood-derived samples (8, 10–14). This is attributable to the fact that CNS tumor ctDNA concentrations are higher in the CSF than in blood, likely due to the blood-brain barrier, which restricts the release of ctDNA into the bloodstream (11).

The feasibility and potential utility of CSF-based liquid biopsy for pediatric and adult CNS tumors is increasingly recognized (8, 13–26). For example, several groups have reported that the H3K27M mutation can be detected with high sensitivity in CSF from patients with diffuse midline glioma (DMG) using digital droplet PCR (ddPCR) or next-generation sequencing (NGS) (8, 16, 20). In medulloblastoma, copy number alterations (CNA) can be detected in CSF, even in patients without radiographic evidence of disease, and the detection of ctDNA after therapy has been shown to correlate with early recurrence (18).

For instance, a study of 45 pediatric and adolescent and young adult (AYA) patients with CNS tumors showed that ctDNA was detected in 46% of CSF samples using targeted capture-based sequencing (22). Similarly, ctDNA was detected in 60% (*n* = 9 of 15) of CSF samples from pediatric patients with CNS tumors with known mutations by targeted capture-sequencing and in 50% (*n* = 11 of 22) of samples from those with known abnormal copy number alterations (CNA) by low pass-whole genome sequencing (LP-WGS) (21). Despite these promising findings, there remains a paucity of studies evaluating the clinical utility of liquid biopsy in a prospective manner across a broad spectrum of pediatric CNS tumors, encompassing various clinical settings and disease stages.

Here, using a combination of liquid biopsy platforms, we analyzed 148 CSF samples from 120 pediatric patients with CNS tumors and assessed the utility of liquid biopsy in various clinical contexts, including initial diagnosis, disease monitoring, detection of recurrence versus second tumor, minimal residual disease, and tumor evolution. Our experience underlines the value of liquid biopsy for pediatric CNS cancer and provides insights for its use in clinical practice and for future research.

## Results

### Patient Cohort and sample characteristics

**Figure 1:**
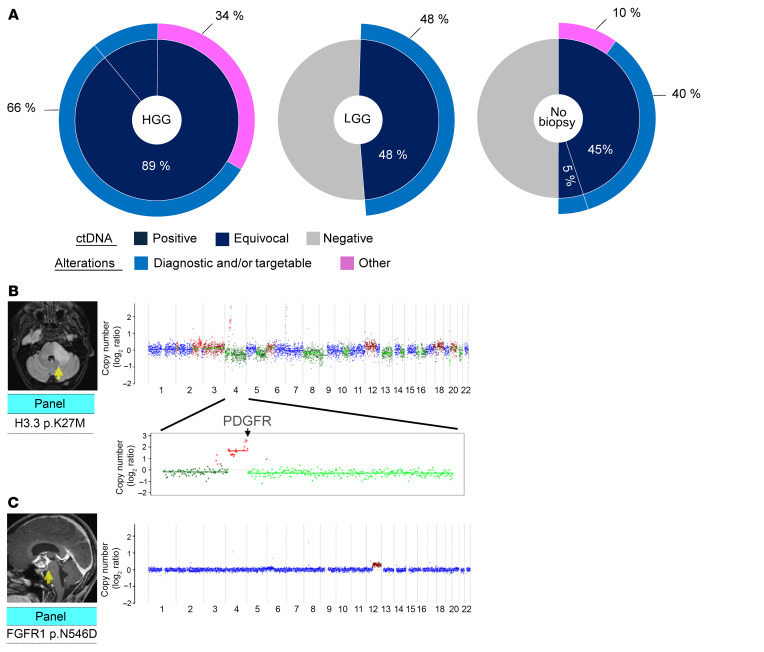
Liquid biopsy at diagnosis of pediatric gliomas. (**A**) The pie charts show the proportion of samples with detectable circulating tumor DNA (ctDNA) in high-grade gliomas (HGG), low-grade gliomas (LGG), and nonbiopsied lesions. The accompanying donut chart depicts diagnostic and/or targetable molecular alterations identified in these samples. (**B**) H3.3 p.K27M and copy number alterations including PDGFRA amplification detected in a patient with nonbiopsied midline lesion, leading to the diagnosis of DMG, H3 K27-altered. (**C**) FGFR1 p.N546D and gain of 12q were detected in a patient with nonbiopsied tumor, which led to the diagnosis of LGG and treatment with targeted therapy.

Between April 2017 and July 2024, we collected 148 CSF samples from 120 patients with pediatric-type brain tumors, along with 32 additional CSF samples that were used as negative controls. Samples were tested with one or more of the following assays: ddPCR for BRAFV600E and/or H3.3K27M, panel NGS, and/or LP-WGS (Supplemental Figure 1C; supplemental material available online with this article; https://doi.org/10.1172/JCI197391DS1). Assays were chosen based on availability at the time of sample collection, as these methods were developed sequentially, and on clinical or sample criteria. For example, patients with tumors characterized by frequent copy number alterations were tested with LP-WGS, whereas samples with limited amount of ctDNA and suspected BRAFV600E or H3K27M mutations were profiled by ddPCR. Overall, 54% (80 of 148) of samples were positive for ctDNA by at least one assay, including one case with a H3K27M mutation detected below the threshold of quantification (equivocal). Six samples were tested exclusively by ddPCR, while 142 samples were tested by NGS; of these, 9 were tested with both ddPCR and NGS (Figure 1C). Regarding clinical context, 67 samples were collected at initial presentation, 25 during treatment, 9 after therapy, and 36 at relapse or progression; the remainder were collected at autopsy or at unknown clinical status. Underlying tumor diagnoses included high grade glioma (*n* = 36), low-grade glioma (*n* = 48), medulloblastoma (*n* = 29) and others (*n* = 15), consisting of pineoblastoma (*n* = 6), ependymoma (*n* = 5), teratoma (*n* = 1), CNS neuroblastoma (*n* = 1), choroid plexus carcinoma (*n* = 1), and glioma NOS (*n* = 1). Additionally, 20 samples were collected from patients with undetermined intracranial lesions, suspicious brain neoplasms, or clinical/radiological diagnoses of CNS tumors without pathologic confirmation, including 5 samples from young adults (32–45 years old) with presumed pediatric type tumors. Thirty-seven plasma samples (including 13 negative controls) were also collected and tested with ddPCR for comparison with CSF samples (Supplemental Figure 1, A and B).

For samples from pediatric patients that underwent sequencing analysis, the median age at the time of sample collection was 9 years (range 0–19 years), and the median volume of CSF collected for cfDNA extraction was 5 ml (range 1–10 ml). Median concentrations of total DNA and cfDNA were 5.25 ng/ml (range 1.3–4,545 ng/ml) and 0.15 ng/ml (range 0.01–124.8 ng/ml), respectively. Both, total DNA and cfDNA concentrations were higher in CSF collected from the ventricles (via intraoperative sampling or external ventricular drains) compared with lumbar puncture (LP) samples (median total DNA 16.25 versus 4.15 ng/ml, *P <* 0.0001; cfDNA 0.62 versus 0.12 ng/ml, *P* = 0.0304) (Supplemental Figure 2A). In addition, we analyzed 8 control ventricular CSF samples from patients with nonneoplastic disorders (e.g., congenital hydrocephalus). Median total DNA and cfDNA concentrations in these controls were 3.2 ng/ml (range: 1.8–315.5 ng/ml) and 0.12 ng/ml (range: 0.02–3.6 ng/ml), respectively. While DNA concentrations were significantly higher in patients with CNS tumors compared with controls (*P* = 0.0192), cfDNA concentrations in ventricular samples did not differ significantly between the 2 groups (*P* = 0.238). These findings highlight the variability in cfDNA levels in CSF and underscore the challenges of interpreting cfDNA quantification in this context (Supplemental Figure 2B).

### ddPCR versus NGS for CSF liquid biopsy

A major question arising for clinical implementation of liquid biopsy in neuro-oncology is determining the most appropriate assay. Several methods are available for analyzing ctDNA; however, in the context of CSF-derived samples, the 2 technologies currently used in clinical settings are ddPCR and NGS, the latter encompassing both deep targeted panel sequencing and/or LP-WGS. We therefore evaluated the relative performance characteristic of these 2 assays in a large cohort of pediatric brain tumors. We first assessed the utility of ddPCR for detecting tumor-specific mutations in cfDNA from CSF or plasma in a subset of patients. To evaluate this approach, we developed ddPCR assays targeting BRAFV600E and H3.3K27M alterations. These targets were chosen, as these are hotspot mutations commonly found in CNS midline gliomas that are difficult to access surgically and may be amenable to targeted therapy (e.g., BRAF V600E) and thus served as proof of principle for the utility of CSF-based liquid biopsy to make diagnoses and enable targeted therapeutics without the need of a surgical biopsy. We tested 17 plasma and 5 CSF samples from patients with BRAFV600E-mutant gliomas (2 HGG; 20 LGG) and 7 plasma and 4 CSF samples from H3.3K27M-mutant diffuse midline gliomas (DMGs). The mutation detection rate from CSF was 80% (4 of 5) for BRAFV600E and 100% (4 of 4) for H3.3K27M, while rate of detection in plasma samples was significantly lower at 0% (0 of 17) for BRAFV600E and 14% (1 of 7) for H3.3K27M. No false positives were identified in negative controls (37 CSF and 11 plasma samples for BRAFV600E; 20 CSF and 6 plasma samples for H3.3K27M), all of which yielded negative results. Consistent with findings from other studies, the detection rate of CNS tumor ctDNA was significantly higher in CSF than in plasma, leading us to focus on CSF-based analyses for NGS testing (14, 27). Although our small cohort demonstrated excellent detection rates using ddPCR, its application is limited by its design as a single mutation test and is therefore not applicable to a range of CNS cancers, particularly in clinical settings where validation of multiple individual assays is impractical.

We next evaluated an NGS-based liquid biopsy approach using a combination of targeted panel sequencing and/or LP-WGS to detect a broad spectrum of genomic alterations, including single nucleotide variants (SNVs), fusions, and copy number alterations (CNAs). Results from cfDNA sequencing were compared with known somatic alterations when tumor profiling was available. A total of 142 samples were sequenced, including 112 samples analyzed with our targeted panel and 92 samples with LP-WGS. Overall, the median sequencing depths of the targeted panel and LP-WGS were 190.2 (0.2–190) and 0.18 (0.01–5.73), respectively. ctDNA was detected in 53% (75 of 142) of samples across the entire cohort and in 58% of samples (69 of 119) with radiologic evidence of disease. Among the latter group, 68% (34 of 50) of intraventricular samples tested positive, compared with 50% (32 of 65) of LP samples. The LP group included 2 equivocal cases with H3.3 K27M mutations where VAF was below the assay’s limit of detection.

The detection rate of ctDNA varied by tumor grade. Among imaging-positive cases, high-grade tumors (WHO grade 3 and 4) were more likely to yield positive samples when compared to low-grade tumors (WHO 1 and 2) with detection rates of 74% versus 48%, respectively.

In HGG, ctDNA was positive in 76% (26 of 34) of samples. Of 48 mutations identified, 7 mutations in 4 samples were uniquely detected by liquid biopsy. One sample yielded mutations compatible with resected tumor profiles but fell below the confidence threshold for positive calling (i.e., equivocal); Targetable mutations were identified in 15 samples, these included alterations such as BRAFV600E and oncogenic mutations in *PDGFRA, IDH, FGFR,* and *PIK3C*. For LGG, we analyzed 43 samples from 39 patients, with an overall positivity rate of 51% (22 of 43). All alterations detected in low-grade gliomas were targetable, including BRAF and FGFR mutations or fusions (Supplemental Table 1).

Overall, similar to previous reports, our experience using liquid biopsy in the pediatric brain tumor population shows high sensitivity for patients with high-grade tumors. For low-grade gliomas, sensitivity is improved with ventricular sampling over LP. ddPCR shows superior sensitivity to NGS-based techniques and is successful with lower cfDNA inputs; however, it is limited by the narrow range of alterations testable by this method.

### Clinical utility of liquid biopsies

Liquid biopsy has a broad range of potential clinical applications, including noninvasive diagnosis (replacing surgical biopsy), monitoring for response to therapy, and early relapse detection, among others. We used our prospectively collected cohort to test the potential utility in these clinical scenarios for the pediatric CNS tumor population.

#### Liquid biopsy for detection of diagnostic alterations.

A total of 67 samples were collected at the time of initial diagnosis, of which 53 had paired tissue available for comparison of molecular alterations. ctDNA in keeping with the primary diagnosis was detected in 60% (*n* = 32 of 53, 1 equivocal). The detection rate for HGG and LGG were 100% and 48%, respectively (Figure 1A). In 45% (17 of 38) of HGG and LGG cases, an actionable alteration was identified (Supplemental Table 1). Among medulloblastoma samples collected prior to therapy, 6 of 8 were obtained via LP and showed positive ctDNA. In 4 samples for which matched cytology was available, it was negative, suggesting higher sensitivity of liquid biopsy in this group of tumors.

In addition, for 14 patients in our cohort, CSF was sampled with the aim of establishing a diagnosis without requiring a surgical procedure. CSF was also collected from 6 additional patients with presumed CNS malignancies during the course of their disease. ctDNA was detected in 50% (10 of 20) of these cases (Figure 1A). Positive ctDNA enabled diagnosis of glioma without biopsy and pointed to potential therapeutic targets. For example, in one case (NA11-D), CNAs, including *PDGFRA* amplification as well as H3.3 K27M SNV, were detected, confirming the clinical-radiologic diagnosis of diffuse midline glioma (DMG) H3 K27-altered and providing a potential therapeutic target (Figure 1B). In another case (ND8-D), FGFR1 N546D and 12q gain were identified, consistent with the diagnosis of a low-grade glioma and leading to consideration of targeted therapy (Figure 1C). In a third case, we detected BRAFV600E using ddPCR (chosen because of low cfDNA quantity) on an LP sample from a 2-year-old male child with presumed midline low-grade glioma (LGG). The detection of BRAFV600E in CSF guided the initiation of targeted therapy with a BRAF inhibitor, resulting in major clinical and radiological improvement. At the last follow-up, 2 years after treatment, the patient remained clinically stable (Supplemental Figure 3B). Conversely, negative ctDNA results were equally informative. In 4 patients with negative ctDNA, the intracranial lesions were stable with median follow-up period from first abnormal imaging of 6.5 (2–7) years. Thus, absence of ctDNA may suggest clinically nonneoplastic or low-grade etiology.

#### Liquid biopsy for evaluation of recurrent or progressive disease.

**Figure 2:**
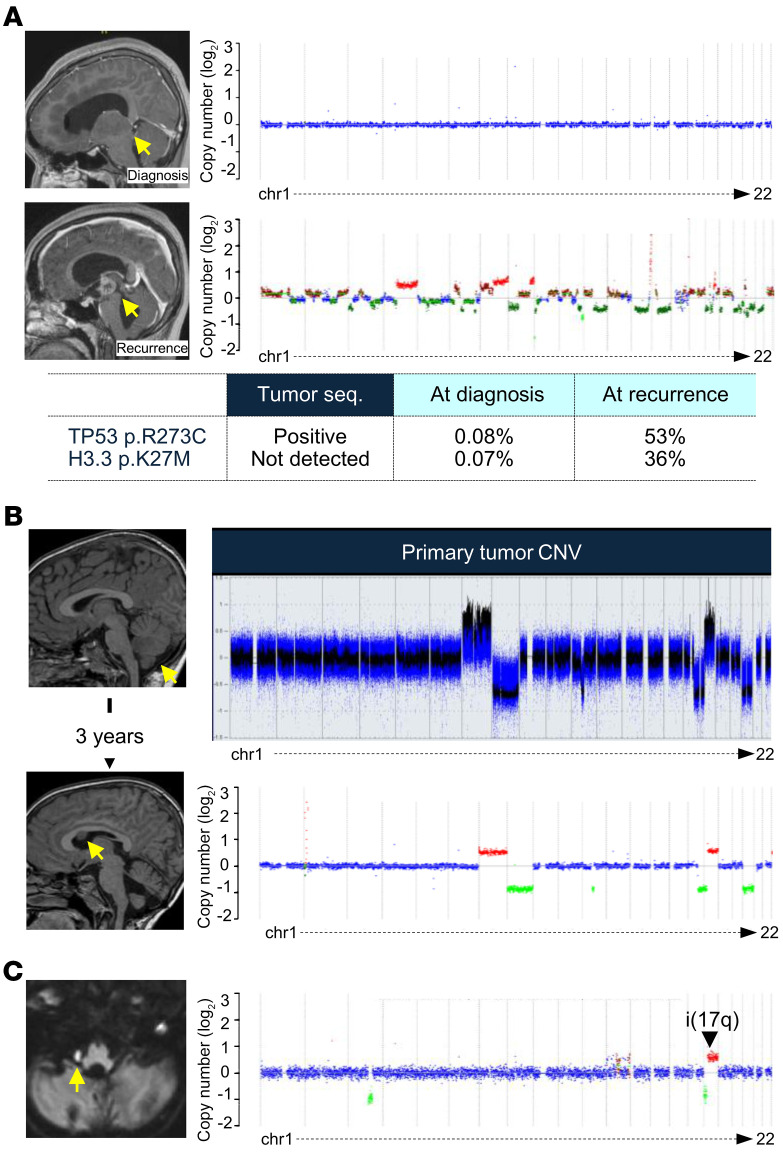
Liquid biopsy at recurrence and progression. (**A**) Diffuse midline glioma. CSF collected at progression reveals H3.3 p.K27M and TP53 p.R273C at higher variant allele frequencies along with CNAs not present at diagnosis. (**B**) Copy number alterations in CSF at recurrence were compatible with CNA detected in the primary tumor, leading to the diagnosis of recurrent MB. (**C**) Detection of CNA including isochromosome 17q leading to diagnosis of a recurrent MB.

In 39 patients, liquid biopsy was done at relapse or progression to distinguish recurrent primary tumor versus secondary neoplasm, to characterize possible tumor evolution, and/or to define radiologically ambiguous lesions. In patients with medulloblastoma, liquid biopsy helped distinguish recurrence from secondary gliomas. Among 5 cases, diagnostic CNAs consistent with recurrent medulloblastoma were detected in 60% (3 of 5). For example, in a suspected recurrent medulloblastoma case (MB13-R), liquid biopsy confirmed recurrence by detecting CNAs consistent with the primary tumor (Figure 2B). In 2 additional cases (MB11-R, MB12-R), loss of 17p and gain of 17q identified by liquid biopsy suggested isochromosome 17q (i(17q)), confirming recurrence and guiding therapy (Figure 2C).

In one DMG case, liquid biopsy at progression identified H3K27M as well as a *TP53* mutations not detected at diagnosis, refining the diagnosis and confirming disease progression (Figure 2A). In another DMG case, liquid biopsy revealed subclonal evolution with *PIK3CA* mutations (Q546R and E545K) that were not present in the primary tumor identified in mutually exclusive reads (Supplemental Figure 3C).

These findings suggest that liquid biopsy may complement traditional diagnostic methods and provide actionable insights in ambiguous or challenging cases.

#### Liquid biopsy for disease monitoring.

**Figure 3:**
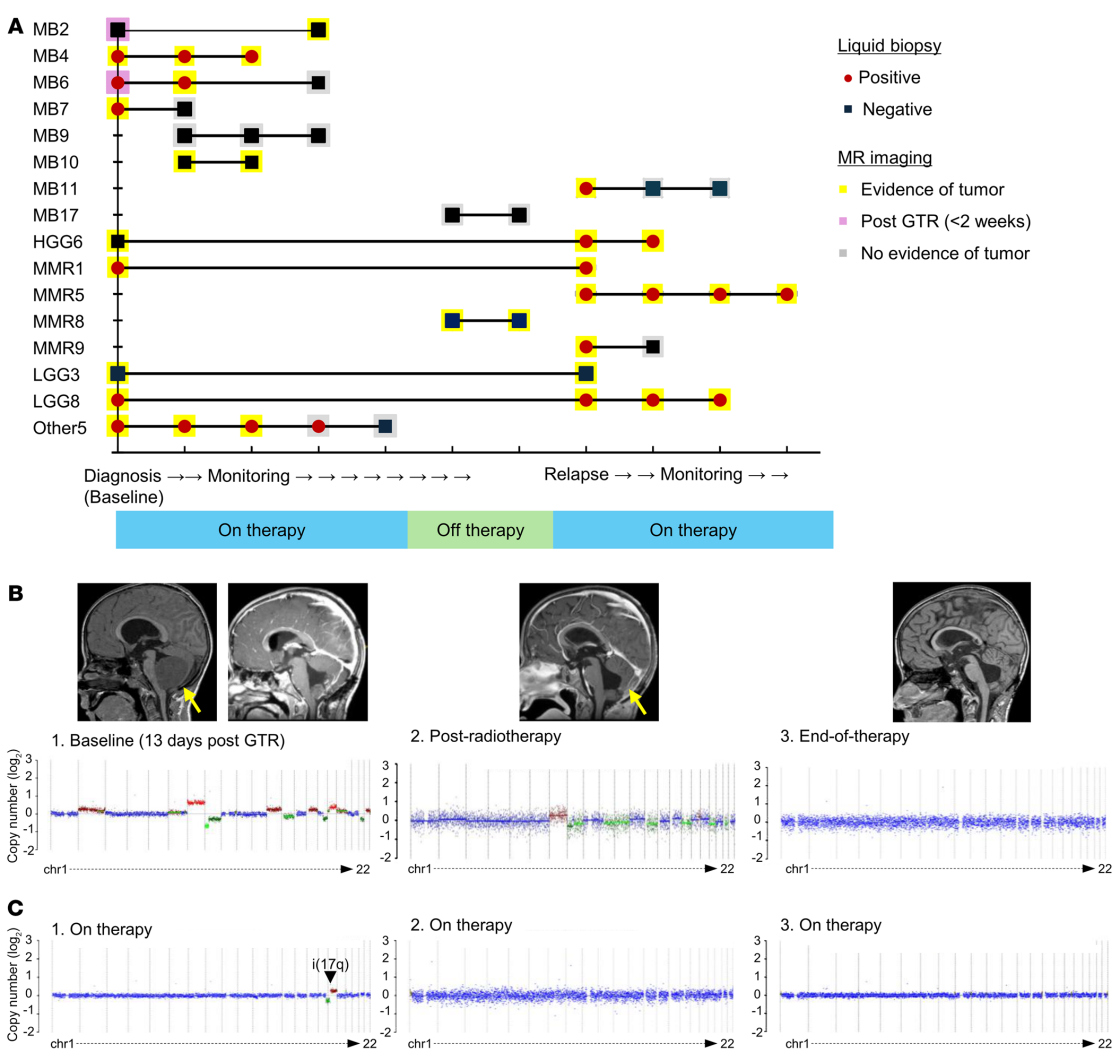
Liquid biopsy during and/or after completion of therapy. (**A**) Positivity of liquid biopsy and evidence of tumor by imaging at the time of sample collection. (**B**) ctDNA was positive after gross total resection (GTR) of medulloblastoma and after radiotherapy, becoming negative at the end-of-therapy (MB6). (**C**) Detection of i(17q) confirmed the existence of recurrent medulloblastoma and ctDNA became undetectable in CSF collected at 15 months and 22 months of therapy (MB11).

To evaluate the ability of our liquid biopsy assay to complement conventional methods such as imaging and CSF cytology for response assessment and disease monitoring, we serially analyzed a total of 43 CSF samples from 16 patients with various tumor types, including 8 medulloblastomas, 7 gliomas, and 1 pineoblastoma. (Figure 3A). Serial samples were collected through lumbar puncture or ommaya reservoir when possible.

Among these, 4 became negative through the course of treatment. Importantly, in one case of infant pineoblastoma, serial liquid biopsy revealed persistent ctDNA positivity throughout induction and high-dose chemotherapy, despite no radiographic evidence of disease and negative CSF cytology. This result prompted continuation of therapy with a maintenance regimen consisting of low-dose oral chemotherapy and biweekly intrathecal (IT) topotecan. After 6 weeks of maintenance therapy, ctDNA levels became undetectable, with no evidence of CNAs. The patient remained clinically and radiologically clear at 6 months following high-dose chemotherapy (Figure 3A, case labeled “Other5”).

In a case of recurrent medulloblastoma (MB011) diagnosed by liquid biopsy, therapy consisting of oral and intrathecal chemotherapy was initiated. ctDNA was undetectable in follow-up samples collected at 15 and 21 months, with no evidence of disease on MRI. This prompted deescalation of therapy, illustrating how serial ctDNA analysis can guide treatment decisions with greater specificity and sensitivity than conventional imaging (Figure 3C).

In addition, liquid biopsy was able to clarify ambiguous radiographic findings in a case of Group 4 medulloblastoma (MB6). MRI after the completion of radiotherapy showed small lesions in the tumor bed and spine of unclear nature; liquid biopsy detected CNAs, ascertaining the presence of residual disease (Figure 3B). Subsequent testing at the end of therapy showed no ctDNA, corroborating MRI findings of complete tumor resolution.

Collectively, these results demonstrate that liquid biopsy-based ctDNA analysis can complement radiological assessments of therapy response, offering superior specificity and sensitivity in monitoring disease activity and informing clinical management.

### Is liquid biopsy in pediatric low-grade gliomas useful?

The use of liquid biopsies and their clinical utility for pediatric LGG has been controversial, to address this gap we profiled 45 CSF samples collected from 42 patients with diagnosis of LGG. Overall 51% (23 of 45) of samples were positive and all alterations found were considered targetable with direct impact for potential therapeutic strategies. Most samples were ventricular, collected either intraoperatively or through external shunts (*n* = 32). Of 32, 18 samples (56%) were ctDNA positive; only 3 of these cases showed leptomeningeal dissemination on imaging. In contrast, lumbar puncture (LP) samples (*n* = 13) had a lower positivity rate, with 5 of 13 (38%) ctDNA-positive, all from patients with leptomeningeal dissemination. Among LP samples, 71% (*n* = 5 of 7) collected from patients with disseminated lesions were positive for ctDNA, whereas none (*n* = 0 of 6) of those collected from patients without disseminated lesions or lesions adjacent to the ventricles were positive. Additionally, 3 samples obtained from cystic lesions were also positive. Detection rates were overall higher in samples from disseminated LGG (80%, 8 of 10) compared with nondisseminated cases (43%, 15 of 35). Notably, all ctDNA-positive samples in nondisseminated LGG were collected via ventricular sampling, with none of the 6 LP samples from nondisseminated tumors testing positive and only 2 with a sequencing depth of greater than or equal to 20×. These findings suggest that ctDNA detection in nondisseminated LGG, particularly when CSF is collected via LP, remains challenging. However, liquid biopsy was able to provide additional molecular insights in cases where tumor sequencing failed or did not detect specific mutations (Supplemental Figure 3A).

### Liquid Biopsy for patients with MMRD: ctDNA detection and MMRDness

In cancer predisposition syndromes such as mismatch repair deficiency (MMRD), liquid biopsies may play an important role in cancer surveillance, diagnosis, and therapeutic monitoring. MMRD patients frequently develop CNS tumors during childhood, particularly high-grade gliomas (HGG) (28, 29). In our cohort, 18 CSF samples from 12 patients with MMRD were analyzed.

**Figure 4:**
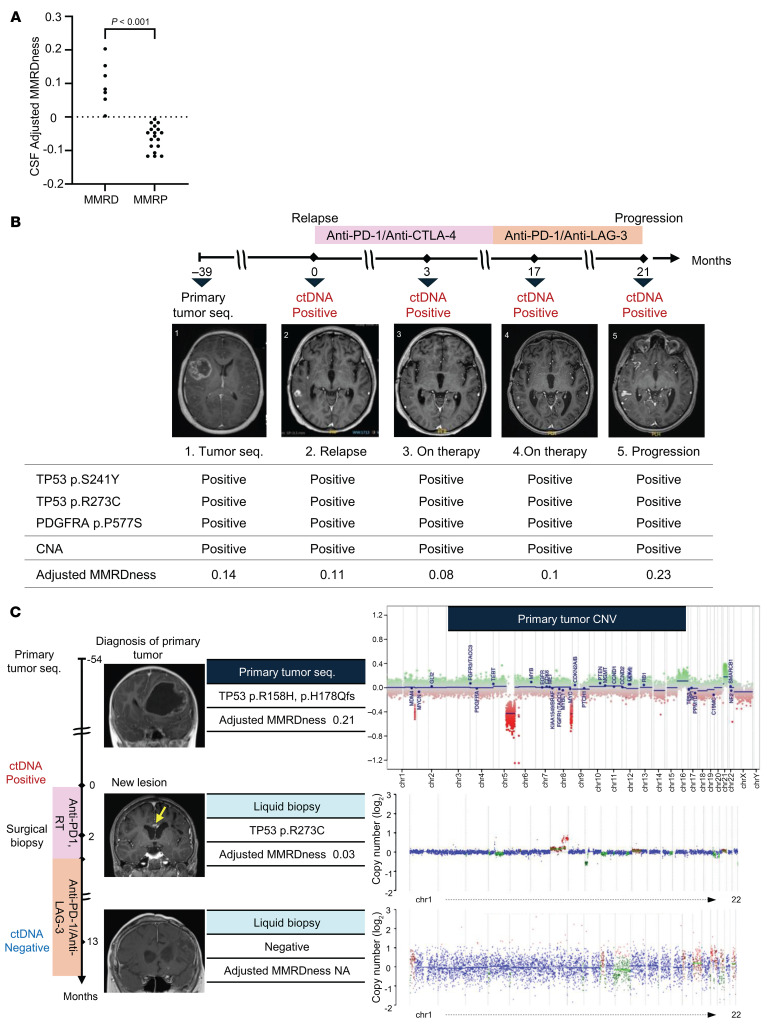
Liquid biopsy in patients with mismatch-repair deficiency. (**A**) CSF samples from patients with MMRD with detectable ctDNA had significantly higher MMRDness scores than samples from patients with mismatch repair–proficient (MMRP) tumors. (**B**) Mutations, copy number alterations (CNA), and high MMRDness persisted throughout therapy for a high-grade glioma despite radiologic improvement, with progression eventually detected by MRI. (**C**) In a patient with high-grade glioma and a new lesion, liquid biopsy identified CNA and 12 variants,including pathogenic variants (PV) and variants of uncertain significance (VUS) distinct from those in the primary tumor. This led to the diagnosis of a new tumor rather than recurrence. A high MMRDness score also supported this finding. ctDNA became undetectable with response to therapy.

In addition to our NGS-based strategy for detecting molecular alterations, we applied the previously reported MMRDness tool to evaluate microsatellite instability in cfDNA using LP-WGS (30). Among these 18 samples, 12 had positive cfDNA, 11 were positive for SNVs and 8 for CNAs. MMRDness scores were calculated for 9 samples with LP-WGS coverage greater than 0.2×. ctDNA-positive samples from patients with MMRD (*n* = 7) exhibited significantly higher MMRDness scores compared with ctDNA-positive samples from patients with MMR-proficient (MMRP) patients (*n* = 18) (median: 0.11 [0.03–0.23] vs. –0.025 [–0.09–0.02], *P <* 0.001) supporting the hypothesis that MMRDness could be reliably evaluated from CSF ctDNA (Figure 4A).

In one case (MMR5), ctDNA analysis at relapse detected 3 pathogenic SNVs and CNAs consistent with the primary tumor at diagnosis (Figure 4B). High MMRDness scores further supported the diagnosis of recurrence. Despite partial response to immune checkpoint inhibitors (ICI) with marked leptomeningeal disease improvement, ctDNA and high MMRDness persisted in follow-up samples, suggesting ongoing active disease. Emerging variants in subsequent liquid biopsies indicated tumor evolution and clonal diversity (Supplemental Figure 3D). The patient eventually died 21 months after relapse.

A second case (MMR9) highlighted the role of liquid biopsy in diagnosing new primary tumors. Following remission from a HGG diagnosed 4 years prior, new MRI-detected lesions raised concerns of recurrence (Figure 4C). However, ctDNA findings revealed a distinct molecular profile from the primary tumor, with P53 p.R273C detected instead of the original P53 mutations (p.R158H and p.H178Qfs). Additionally, 12 SNVs detected in the CSF at query relapse were absent from the primary tumor sequencing. High MMRDness (0.03) and disparate CNAs supported the diagnosis of a second tumor in the context of MMRD, rather than a relapse of the primary tumor. Surgical biopsy confirmed these findings, and the patient responded well to radiotherapy and subsequent immunotherapy. Follow-up liquid biopsy at 13 months showed no ctDNA evidence, correlating with clinical and radiological stability at the 32-month follow-up.

These findings demonstrate that liquid biopsy, with ctDNA detection complemented by MMRDness analysis, can aid in distinguishing new tumor from recurrent disease in pediatric patients with MMRD-associated CNS tumors. The ability to detect ctDNA and emerging tumor clones facilitates early cancer detection, may help guide therapeutic decisions and monitor disease evolution. This combined approach holds promise for improving outcomes in this high-risk population.

## Discussion

In recent years, liquid biopsy has emerged as a transformative tool to augment current methods for tumor diagnosis, treatment response assessment, and disease monitoring. In this study, we demonstrate the clinical utility of CSF liquid biopsy in pediatric patients with CNS tumors across diverse clinical contexts. Overall, ctDNA was detected in 54% of CSF samples with positive MRI with higher detection rates in high grade tumors, underscoring its potential as a minimally invasive method to acquire valuable molecular information across multiple time points. Consistent with previous studies, our findings confirm CSF as the preferred sample source for detecting molecular alterations using cfDNA compared with plasma (31).

At diagnosis, liquid biopsy identified diagnostic and/or targetable alterations in nearly 70% of HGG and 50% of LGG cases. Our cohort included a large number of patients with LGG, where ctDNA detection rates were lower, particularly in nondisseminated cases. While ctDNA was detected in 15 of 29 ventricular samples from nondisseminated tumors, no ctDNA was detected in 6 LP samples. In contrast, almost all disseminated cases had detectable molecular alterations in CSF. Additionally, in a patient with a cervicomedullary tumor and no biopsy, a BRAFV600E mutation was detected by ddPCR. This pattern aligns with evidence linking tumor burden and proximity to CSF spaces with ctDNA detectability (7). This discrepancy likely reflects both anatomical sampling factors and the inherent biological characteristics of LGGs, which are known to shed substantially lower amounts of ctDNA than high-grade gliomas (HGGs). This is in keeping with observations from prior studies. For example, Madlener et al. demonstrated improved detection rates in intraventricular CSF versus LP samples, and Miller et al. reported four cases of nondisseminated LGGs, with ctDNA-negative results (22, 32).

While these numbers are small, these findings suggest that lumbar puncture samples in nondisseminated LGG cases may not be ideal for ctDNA analysis using standard sequencing methods, and that alternative strategies, such as intraventricular sampling or more sensitive detection techniques like droplet digital PCR, may be required to improve yield in this context.

At recurrence, molecular profiling provided critical insights into disease etiology and tumor evolution. For instance, liquid biopsy identified a pathognomonic isochromosome 17 in a case of medulloblastoma recurrence 7 years after therapy, confirming relapse. Similarly, in a mismatch repair–deficient (MMRD) patient, distinct molecular profiles differentiated a second malignancy from a suspected recurrence of primary high-grade glioma. These findings underscore the value of CSF liquid biopsy in refining diagnoses and guiding treatment decisions in complex clinical scenarios.

MMRD is diagnosed in up to 9% of pediatric high-grade gliomas, and the identification of this specific tumor subtype is critical, given the remarkable responses to immunotherapy (29). By applying our previously reported MMRDness tool to liquid biopsy, we detected MMRDness signatures in all MMRD tumors with detectable CNAs. This tool offers a minimally invasive method for diagnosing new MMRD tumors, with profound implications for treatment planning (28, 33). Furthermore, CSF liquid biopsy can complement imaging in assessing disease remission and guiding treatment discontinuation or deescalation, particularly when ctDNA is undetectable.

Based on our findings, we propose several recommendations for the use of CSF liquid biopsy in pediatric CNS tumors (Supplemental Figure 4). For patients with HGG, medulloblastoma, or disseminated LGG, we recommend the routine use of NGS-based liquid biopsy, given the high ctDNA positivity rates observed in these tumor types. We recently reported that liquid biopsy is highly sensitive for CNS germ cell tumors as well: CNAs were detected in 89% at diagnosis (26).This approach facilitates noninvasive diagnosis and provides a comprehensive baseline molecular profile for monitoring treatment response and disease progression. For nondisseminated LGG, NGS assays demonstrate higher yields with ventricular samples, as ctDNA was not detected in lumbar puncture samples in this cohort. However, for patients requiring minimally invasive diagnostics, CSF collection via LP can still be utilized, with ddPCR being the preferred method to detect specific targetable variants, such as BRAF V600E. In addition to molecular profiling for mutations, rearrangements and CNAs, the incorporation of MMRDness assessment into liquid biopsy workflows can help identify mismatch repair–deficient tumors. These tumors are particularly important to recognize, as they often respond remarkably well to immunotherapy. By integrating these methodologies, CSF liquid biopsy can serve as a powerful tool for guiding diagnostic and therapeutic strategies, ultimately advancing personalized care for pediatric and patients with AYA with CNS tumors.

This study demonstrates the potential of CSF liquid biopsy as a minimally invasive tool in a real-world clinical setting. By enabling dynamic molecular profiling, liquid biopsy has the capacity to help guide personalized therapy, monitor disease progression, and provide early indicators of treatment response. However, the successful clinical integration of this approach hinges on the standardization and optimization of processes, including sample collection, quality assurance, and data interpretation. The present study is limited by its heterogeneous cohort, encompassing a range of CNS tumor types with samples collected at variable timepoints. Future prospective studies with larger, uniform cohorts and standardized sampling intervals are essential to validate the prognostic and therapeutic utility of this approach. Ultimately, the widespread adoption of liquid biopsy has the potential to redefine CNS tumor management, enabling evidence-based decisions for escalation or deescalation of therapies, minimizing treatment-associated toxicities and improving outcomes for children with brain tumors.

## Methods

### Sex as a biological variable.

Sex was not considered as a biological variable in this study. Our study included both male and female patients.

### Patients and sample collection.

This multiinstitutional study included CSF and plasma samples from patients with confirmed or suspected CNS tumors irrespective of tumor type. Samples were obtained at variable timepoints during therapy between 2017 and 2024. All participants and/or their guardians signed informed consent in accordance with local REB (SickKids, Toronto, Canada-REB #1000071241). CSF samples were obtained via lumbar puncture, intraoperatively, from external ventricular drains (EVD) or from Ommaya reservoirs. CSF (1–10 ml) was collected into plain sterile collection tubes (if samples were in-house and could be processed within 2 hours), otherwise Streck tubes (Streck) were used. Samples that were shipped from external institutions were collected in Streck tubes and stored at room temperature for up to 7 days before cfDNA extraction, as per manufacturer recommendations (34, 35). To remove cellular debris, CSF was centrifuged at 1,000*g* for 10 minutes at room temperature; supernatant was stored at –80°C until cfDNA extraction. Blood samples were collected in EDTA tubes and processed within 2 hours of collection. Samples were centrifuged at 820g for plasma separation; supernatants were further centrifuged at 2000g to remove cellular debris, supernatants were aliquoted into 1.5 ml Eppendorf tubes and stored at –80°C until use.

### Extraction of Cell-Free DNA.

CSF samples underwent cfDNA extraction using QIAamp Circulating Nucleic Acid Kit (Qiagen) according to the manufacturer’s protocol; cfDNA was eluted in 60 μL of AVE buffer and stored at –20°C until the library preparation. DNA was quantified using Qubit dsDNA Quantification assay (High sensitivity) (Thermo Fisher) and cfDNA (75–300 bp) was quantified using Cell-Free DNA screentape assay, by TapeStation (Agilent Technologies).

### Droplet digital PCR.

ddPCR was performed using the RainDrop Digital PCR system (Bio-Rad) according to manufacturer’s instructions. Primers and probes were specifically designed to detect BRAFV600E (BRAF c.1799T>A) and H3.3K27M (H3-3A c.83A>T) mutations in separate reactions. To ensure optimal sensitivity, cfDNA was pre-amplified using high-fidelity master mix under the following conditions: 95°C for 30 seconds, followed by 15 cycles of 95°C for 10 seconds and annealing temp for 15 seconds; 72°C for 5 seconds with final extension at 72°C for 2 minutes. Subsequently, 1 μl of pre-amplified PCR product was taken into the ddPCR reaction. The ddPCR analysis was conducted using the RainDrop Analyst II software, and the positivity threshold was determined based on Poisson statistics. The assay’s performance characteristics were validated using serially diluted formalin-fixed, paraffin-embedded FFPE samples with the respective mutations described. This validation demonstrated reliable detection of variant allele frequencies (VAF) as low as 0.10% with a minimum DNA input of 1.0 ng. This high sensitivity underscores the utility of this assay for detecting low-frequency variants in samples with low content of cfDNA.

### Library preparation and Low-Pass Whole Genome Sequencing.

A maximum of 30ng of cfDNA was used as input for library preparation. The median input amounts were 19.4 ng (range 5.8–2376 ng) of total DNA as measured by Qubit (Thermo Fisher) and 0.51 ng (range 0.06–30 ng) of cfDNA as measured by Tapestation (Agilent Technologies). End repair, A-tailing and ligation of unique molecular identifiers (UMIs) were performed using TruSight Oncology 500 ctDNA v2 (Illumina). Universal dual index (UDI) adapter, purification, and target enrichment for panel sequencing were constructed using Twist library preparation kits according to the manufacturer’s protocols (Twist Bioscience). Library quality and quantity were assessed using the 2100 Bioanalyzer system (Agilent Technologies). Sequencing was conducted on the NextSeq 500/550 (Illumina) with 150-base paired-end reads. For broad genomic profiling and detection of CNA, Low-Pass Whole Genome Sequencing (LP-WGS) was performed targeting a depth of 1–3× coverage.

### Targeted panel sequencing.

A custom 21 gene panel targeting commonly altered glioma genes was designed (*BRAF, CDKN2A, EGFR, FGFR1, FGFR2, FGFR3, H3F3A, HIST1H3B, HIST1H3C, IDH1, IDH2, KRAS, MYB, MYL1, MYC, MYCN, PDGFRA, PIK3CA, PTPN11, TERT, TP53*). Probes covered all exons and key introns targeting common fusion breakpoints, spanning a total target size of 81 kilobases. The panel was synthesized by Twist Bioscience, and hybrid capture was performed according to the manufacturer’s recommendations. A maximum of 30 ng or 50 ul of cfDNA was used as input for library preparation. Libraries were pooled and the hybridized with capture probes for 18-21 hours, the final enriched libraries were then PCR-amplified, cleaned and sequenced with 150 bp paired end on the Nextseq instrument. Panel sequencing aimed to achieve 10,000-20,000x raw read depth, with a minimum UMI-corrected read depth of 20 x established as the cutoff for reliable detection of VAFs ≥ 10%.

### Panel Validation and performance characteristics.

We validated the performance characteristics of our custom hybridization panel using the Twist cfDNA Pan-Cancer Reference Standard, which includes 34 variants of interest expected to be detectable by our assay. Variants were tested at varying variant allele frequencies (VAFs), ranging from 0% (WT) to 5%. Sequencing was performed to a target depth of 20,000× raw reads, with 10 ng of DNA input. The assay demonstrated 100% sensitivity (34/34) for detecting variants with VAFs > 0.5%–5%. At a VAF of 0.5%, 97% of variants were detected. However, sensitivity declined for VAFs below 0.5%, with detection rates of 50% for VAFs of 0.2% and 0.1%. Based on these results, the limit of detection (LOD) was established at 0.5% VAF with a minimum cfDNA input of 10 ng.

### Bioinformatic analysis.

Raw sequencing reads were analyzed using the UMI-aware DRAGEN v3.10.4 pipeline in the BaseSpace platform (Illumina; https://cac1.sh.basespace.illumina.com) to detect SNV/indels and fusions. Variants were interpreted with BaseSpace variant interpreter v.2.17; variants were annotated with COSMIC and ClinVar databases, while alterations recorded in population databases (i.e., >0.01 allele frequency in 1000 Genomes, TOPMed, 1000 Genomes project, and NHLBI Exome Project) were excluded. Threshold for mutation calling included (1) VAF is ≥0.5%, based on our validation assay (described above, Figure S1B), (2) mutant allele had >2 reads, and (3) depth of variant and wild-type allele is ≥20. Fusion genes were additionally detected using Arriba. Automatically called pathogenic variants, fusions and clinically relevant canonical fusion such as *KIAA1549:BRAF* were checked manually on IGV. All alterations were manually reviewed by at least one individual. Reviewers were not blinded to clinical or tumor data. In cases where no alterations were automatically detected, sequencing data were manually examined for mutations previously identified in the tumor and/or suspected based on the clinical differential diagnosis. In addition, all cases were reviewed in a group multi-disciplinary rounds-type setting and a consensus agreement on the presence or absence of ctDNA was reached. Mean panel coverage <20 was considered to be inadequate sequencing. When hot-spot mutation/fusion relevant to diagnosis or known by matched tumor sequencing were present on IGV below the established thresholds, we considered the results equivocal. Copy number analysis was performed using ichorCNA (25). Mismatch repair deficiency scores were calculated from LP-WGS as previously described (30). Briefly, BAM files were analyzed for reads containing insertions or deletions at microsatellites, defined as repeats of five or more nucleotides, using MSMuTect (36).

### Statistics.

Demographic characteristics were described using median values and range when continuous variables; frequencies in percentage were used to describe categorical variables. The Mann-Whitney *U* test was used for comparison between continuous variables and χ^2^ test for categorical variables. Analysis was performed using GraphPad Prism 10.2.2 (GraphPad Software, Boston, USA) and R version 4.0.2, *P* < 0.05 was considered statistically significant.

### Study approval.

This study was approved by the Hospital of Sick Children local research ethics board (SickKids, Toronto, Canada-REB #1000071241) and all participants and/or their guardians signed informed consent prior to participation.

### Data availability.

Sequencing data generated in this study is available at EGA (EGAS50000001528). To comply with data access regulations these data are available under controlled access; requests can be made to the corresponding author.

Values for all data in graphs are reported in the Supporting Data Values file.

## Author contributions

This project was conceptualized by L Nobre, UT, and CH. Clinical data were collected by L Nobre, YN, and IB. Investigations were carried out by L Nobre, YN, MZ, MJ, JS, MR, MK, RY. Analysis, visualization, and manuscript draft preparation was done by L Nobre, YN, RS, IB, UT, and CH. Manuscript editing was done by L Nobre, YN, CH, UT, MZ, MJ, JS, MR, MK, RY, ABL, CLN, AD, CC, SZ, SAC, MM, JC, JB, AB, JL, LS, ME, EB, L Negm, CL, RKL, VR, APYL, AH, UB, and PBD. The order of co–first authors was determined by the timing of their contributions.

## Conflict of interest

The authors have declared that no conflict of interest exists.

## Funding support

- Proof of Principle Grant from The Hospital for Sick Children (to CH).
- The Canadian Institutes of Health Research, #159805 (to CH).
- The Canadian Cancer Society (to CH).
- The We Love You Connie Foundation (to CH).
- Garron Family Cancer Center at SickKids (to CH).
- SickKids Clinician Training Program (to LN).
- Ben Stelter Foundation through the Women and Children’s Health Research Institute (to LN).
- Jeffrey Brock Cancer Genetics Research Fellowship (to YN).
- Garron Family Cancer Center Research Fellowship/Scotiabank Clinician Scientist Fellowship (to YN).
- Atrium/CMCC and Hold’em for Life Oncology Fellowship (to YN).
- Tokyo Children’s Cancer Study Group Scholarship of the Gold Ribbons Network (to YN).

## Supplementary Material

Supplemental data

ICMJE disclosure forms

Supplemental table 1

Supporting data values

## Version 1 06/15/2026

Electronic publication

## References

[1] Hossain MJ (2021). Epidemiology and prognostic factors of pediatric brain tumor survival in the US: Evidence from four decades of population data. Cancer Epidemiol.

[2] Ryall S (2020). Integrated molecular and clinical analysis of 1,000 pediatric low-grade gliomas. Cancer Cell.

[3] Nobre L (2020). Outcomes of BRAF V600E pediatric gliomas treated with targeted BRAF Inhibition. JCO Precis Oncol.

[4] Bouffet E (2023). Dabrafenib plus trametinib in pediatric glioma with *BRAF* V600 mutations. N Engl J Med.

[5] Donson AM (2023). Significant increase of high-risk chromosome 1q gain and 6q loss at recurrence in posterior fossa group A ependymoma: A multicenter study. Neuro Oncol.

[6] Lau LMS (2024). Precision-guided treatment in high-risk pediatric cancers. Nat Med.

[7] Miller AM (2019). Tracking tumour evolution in glioma through liquid biopsies of cerebrospinal fluid. Nature.

[8] Cantor E (2022). Serial H3K27M cell-free tumor DNA (cf-tDNA) tracking predicts ONC201 treatment response and progression in diffuse midline glioma. Neuro Oncol.

[9] Febbo PG (2024). Recommendations for the equitable and widespread implementation of liquid biopsy for cancer care. JCO Precis Oncol.

[10] Pan W (2015). Brain tumor mutations detected in cerebral spinal fluid. Clin Chem.

[11] De Mattos-Arruda L (2015). Cerebrospinal fluid-derived circulating tumour DNA better represents the genomic alterations of brain tumours than plasma. Nat Commun.

[12] Friedman JS (2022). Tapping into the genome: the role of CSF ctDNA liquid biopsy in glioma. Neurooncol Adv.

[13] Pagès M (2022). Liquid biopsy detection of genomic alterations in pediatric brain tumors from cell-free DNA in peripheral blood, CSF, and urine. Neuro Oncol.

[14] Izquierdo E (2021). Droplet digital PCR-based detection of circulating tumor DNA from pediatric high grade and diffuse midline glioma patients. Neurooncol Adv.

[15] Pentsova EI (2016). Evaluating cancer of the central nervous system through next-generation sequencing of cerebrospinal fluid. J Clin Oncol.

[16] Stallard S (2018). CSF H3F3A K27M circulating tumor DNA copy number quantifies tumor growth and in vitro treatment response. Acta Neuropathol Commun.

[17] Pan C (2019). Molecular profiling of tumors of the brainstem by sequencing of CSF-derived circulating tumor DNA. Acta Neuropathol.

[18] Liu APY (2021). Serial assessment of measurable residual disease in medulloblastoma liquid biopsies. Cancer Cell.

[19] Ronsley R (2024). Detection of tumor-derived cell-free DNA in cerebrospinal fluid using a clinically validated targeted sequencing panel for pediatric brain tumors. J Neurooncol.

[20] Panditharatna E (2018). Clinically relevant and minimally invasive tumor surveillance of pediatric diffuse midline gliomas using patient-derived liquid biopsy. Clin Cancer Res.

[21] O’Halloran K (2023). Low-pass whole-genome and targeted sequencing of cell-free DNA from cerebrospinal fluid in pediatric patients with central nervous system tumors. Neurooncol Adv.

[22] Miller AM (2022). Next-generation sequencing of cerebrospinal fluid for clinical molecular diagnostics in pediatric, adolescent and young adult brain tumor patients. Neuro Oncol.

[23] Kojic M (2023). Efficient detection and monitoring of pediatric brain malignancies with liquid biopsy based on patient-specific somatic mutation screening. Neuro Oncol.

[24] Li D (2021). Standardization of the liquid biopsy for pediatric diffuse midline glioma using ddPCR. Sci Rep.

[25] Adalsteinsson VA (2017). Scalable whole-exome sequencing of cell-free DNA reveals high concordance with metastatic tumors. Nat Commun.

[26] Nakano Y (2024). High detection rate of circulating-tumor DNA from cerebrospinal fluid of children with central nervous system germ cell tumors. Acta Neuropathol Commun.

[27] Bettegowda C (2014). Detection of circulating tumor DNA in early- and late-stage human malignancies. Sci Transl Med.

[28] Das A (2024). Clinical updates and surveillance recommendations for DNA replication repair deficiency syndromes in children and young adults. Clin Cancer Res.

[29] Negm L (2025). The landscape of primary mismatch repair deficient gliomas in children, adolescents, and young adults: a multi-cohort study. Lancet Oncol.

[30] Chung J (2021). DNA polymerase and mismatch repair exert distinct microsatellite instability signatures in normal and malignant human cells. Cancer Discov.

[31] Wang Y (2015). Detection of tumor-derived DNA in cerebrospinal fluid of patients with primary tumors of the brain and spinal cord. Proc Natl Acad Sci U S A.

[32] Madlener S (2025). Detection of H3F3A K27M or BRAF V600E in liquid biopsies of brain tumor patients as diagnostic and monitoring biomarker: impact of tumor localization and sampling method. Acta Neuropathol.

[33] Das A (2023). Efficacy of nivolumab in pediatric cancers with high mutation burden and mismatch repair deficiency. Clin Cancer Res.

[34] Diaz IM (2023). Pre-analytical evaluation of streck cell-free DNA blood collection tubes for liquid profiling in oncology. Diagnostics (Basel).

[35] Wong D (2024). Early cancer detection in li-fraumeni syndrome with cell-free DNA. Cancer Discov.

[36] Maruvka YE (2017). Analysis of somatic microsatellite indels identifies driver events in human tumors. Nat Biotechnol.

