# Supplementary material for: Tumor-derived cell-free DNA detected in cerebrospinal fluid enables minimally invasive profiling of pediatric brain tumors
Source: J Clin Invest. 2026 Jun 15;136(12):e197391. doi: 10.1172/JCI197391 (PMC13262732; doi:10.1172/JCI197391)
Supplement: Supplemental data [file jci-136-197391-s075.pdf]

Supplementary Figure 1

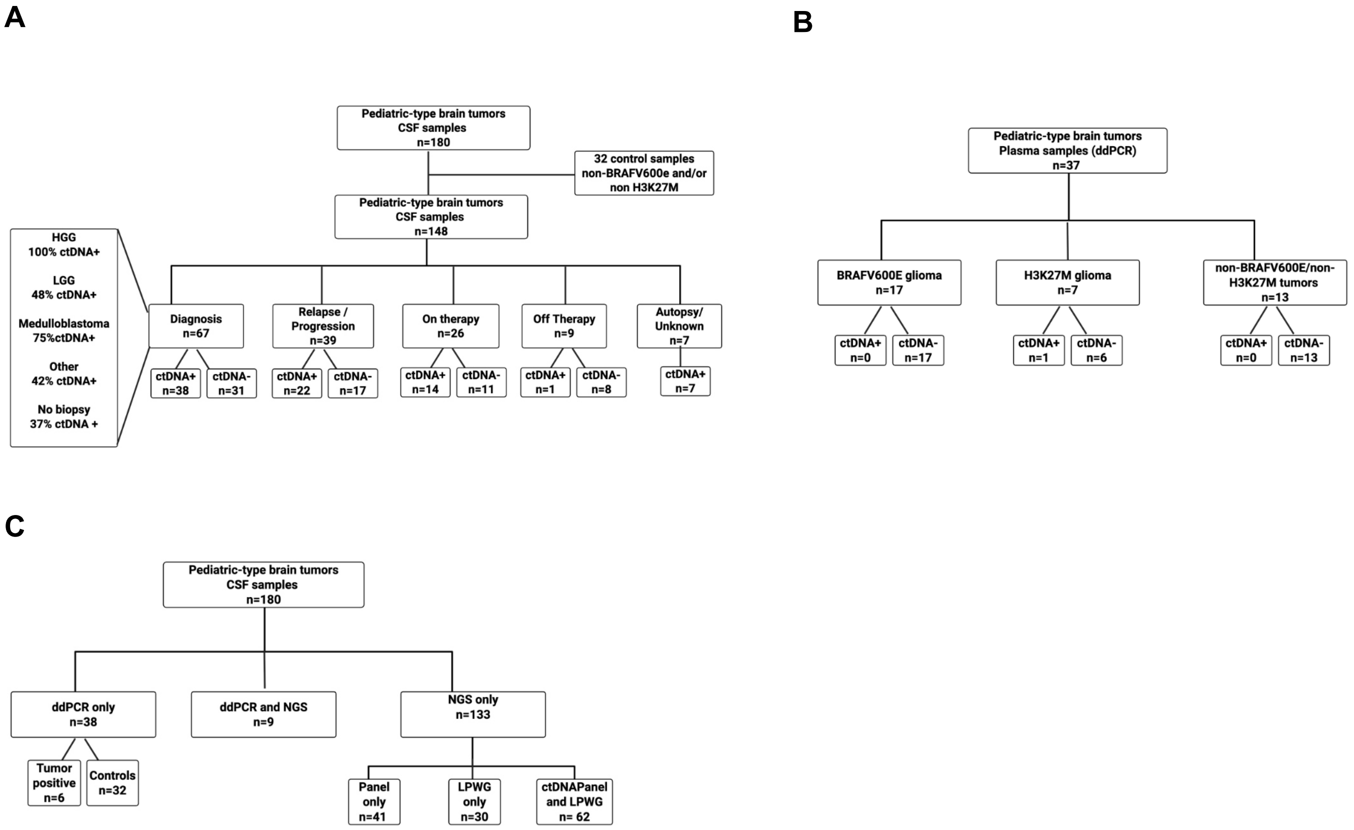

Supplementary Figure 1. Consort diagram describing study cohort and liquid biopsy results. (A) CSF samples(B) plasma samples and (C) assays performed for CSF samples.

Supplementary Figure 2

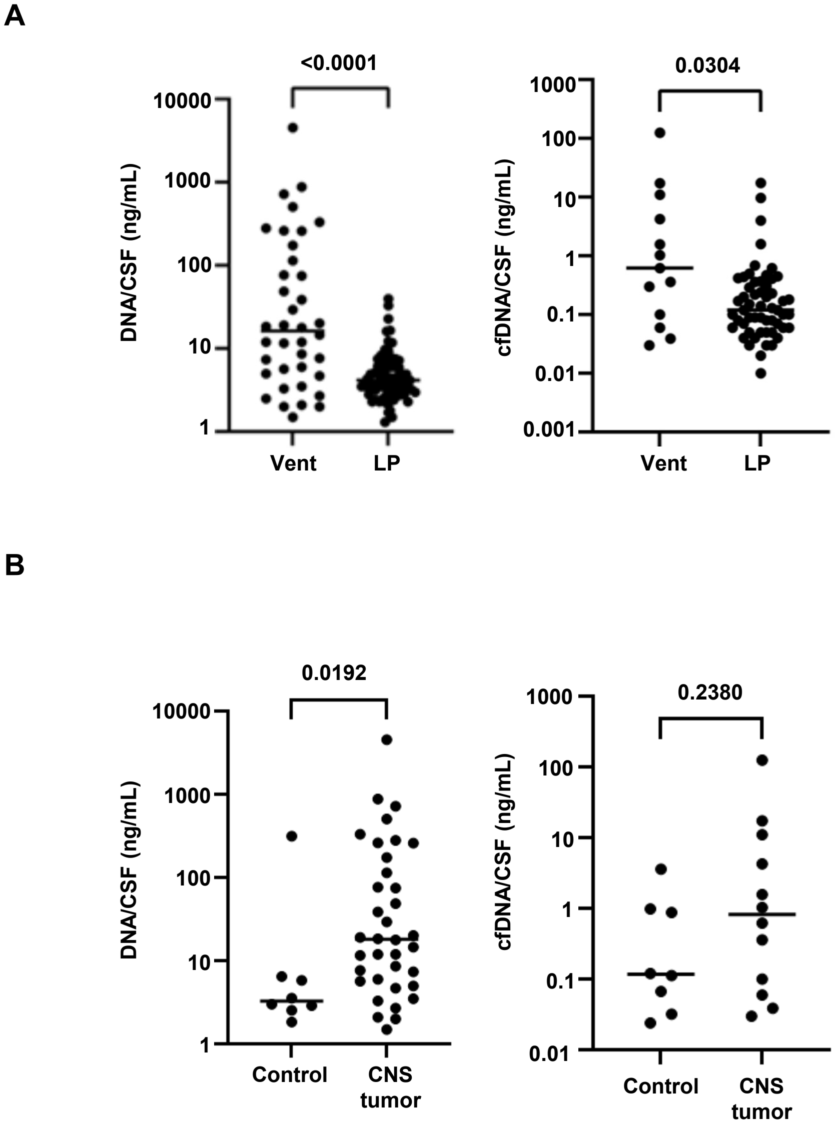

Supplementary Figure 2. Comparison of DNA content between samples. (A) Ventricular samples tend to have higher quantities of total DNA although no statistical significant differences were found for cfDNA. (B) Similarly, samples from patients with CNS tumor have higher total DNA amounts when compared to non-tumor controls.

Supplementary Figure 3

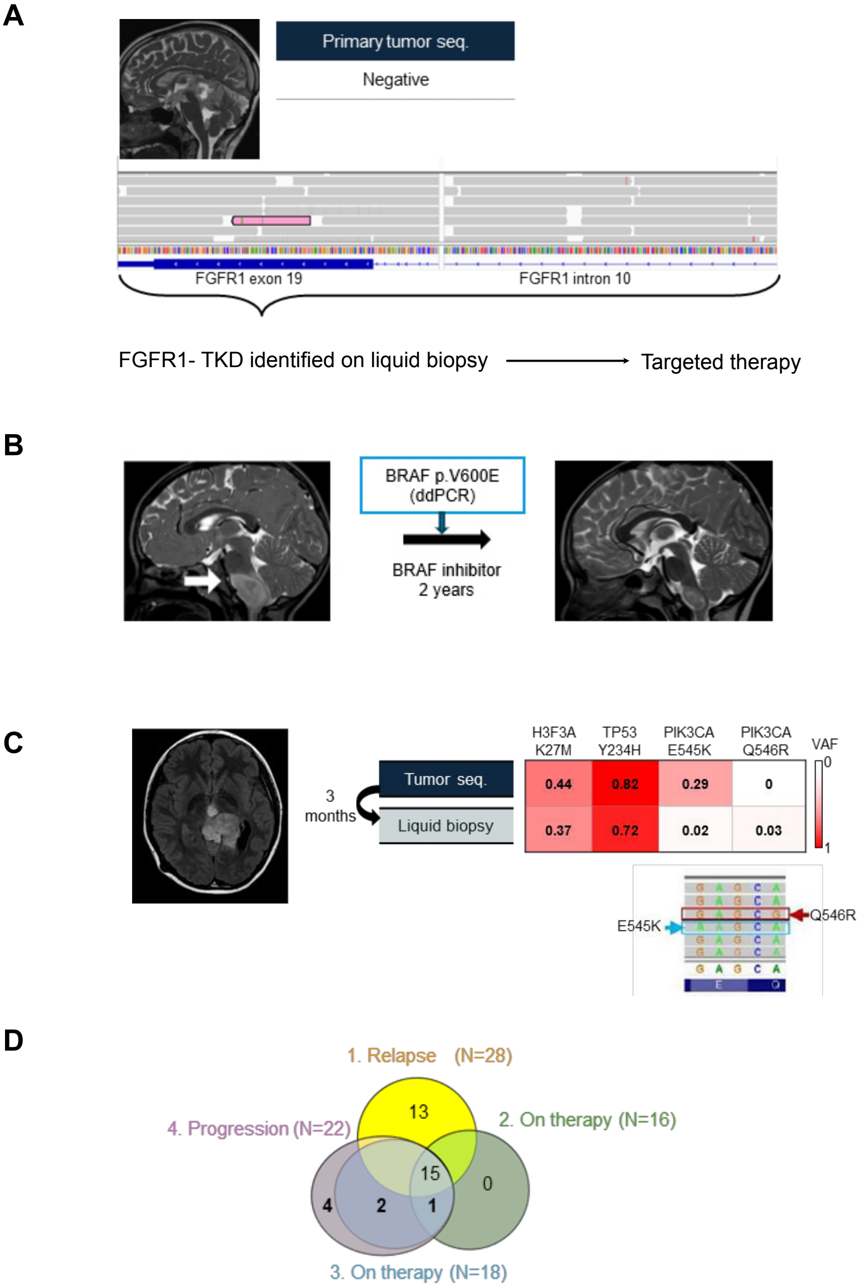

Supplementary Figure 3. (A) Example of a disseminated low grade glioma where molecular alterations were not identified on tumor biopsy, however, panel sequencing of CSF cfDNA identified a FGFR1-TKD and targeted therapy with MEK inhibitor was initiated. (B) Presumed brainstem glioma by imaging with BRAFV600E mutation identified in CSF by ddPCR guiding initiation of targeted therapy with reduction in tumor size.(C) Diffuse midline glioma where sequential CSF samples identified tumor evolution with novel mutation identified. (D) Emerging variants detected in CSF throughout therapy of a patient with MMRD HGG illustrating tumor evolution and clonal diversity.

Supplementary Figure 4

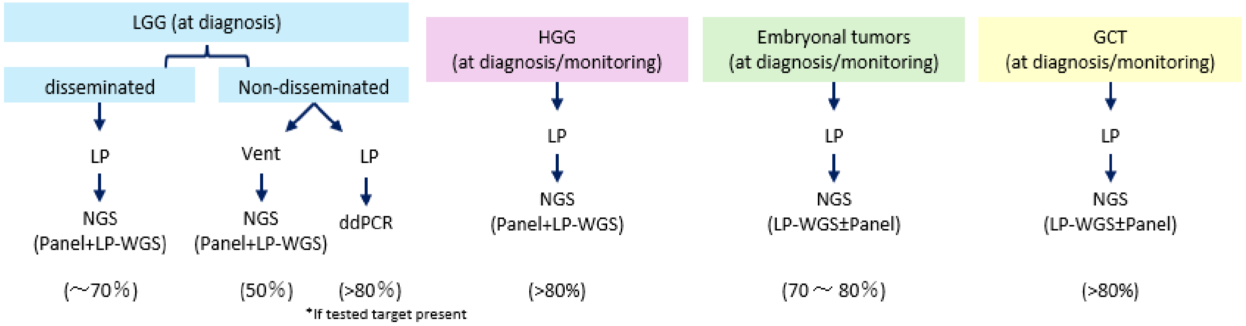

Supplementary Figure 4. Recommendations for liquid biopsy use in pediatric brain tumors according to tumor type and source of CSF.
